# Aortic valve replacement in elderly with small aortic root and low body surface area; the Perceval S valve and its impact in effective orifice area

**DOI:** 10.1186/s13019-016-0438-7

**Published:** 2016-04-11

**Authors:** Panagiotis Dedeilias, Nikolaos G. Baikoussis, Efstathia Prappa, Dimitrios Asvestas, Michalis Argiriou, Christos Charitos

**Affiliations:** Cardiothoracic and Vascular Surgery Department, “Evangelismos” General Hospital of Athens, 45-47 Ipsilantou Street, Kolonaki, Athens, Greece; Department of Cardiology, “Evangelismos” General Hospital of Athens, Athens, Greece

**Keywords:** Perceval S, Aortic valve, Stentless aortic valve, Sutureless valve, Self-expanding valve, Aortic valve stenosis, Heart valve surgery

## Abstract

**Background:**

The aim of this study is to see how the sutureless, stentless, Perceval S aortic valves behave when implanted in elderly patients with small aortic root and the comparison with a second group of patients with similar characteristics where a conventional stented bioprosthesis was implanted. This is a prospective randomized institutional study.

**Methods:**

Our material is composed from 25 patients who underwent aortic valve replacement with sutureless self-anchoring Perceval S valve implantation (LivaNova), compared with 25 patients with conventional stented biological prosthesis implanted (soprano LivaNova group). The two groups of patients have similar demographic and medical characteristics with severe aortic stenosis. The study was conducted from January 2012 to June 2014. Preoperative, intraoperative and postoperative parameters were studied in order to investigate the utility of the Perceval S valves in this group of patients.

**Results:**

The Perceval S valve implantation seems to be an interesting biological valve with good hemodynamic characteristics as compared with the typical biological prosthesis providing shorter ischemia time (40 ± 5.50 min vs 86 ± 15.86 min; *p* < 0.001), shorter extracorporeal circulation time (73.75 ± 8.12 min vs 120.36 ± 28.31 min *p* < 0.001), less operation time (149.38 ± 15.22 min vs 206.64 ± 42.85 min; *p* < 0.001) and better postoperative recovery. The postoperative gradients were 23.5 ± 19.20 mmHg vs 24.5 ± 19.90 mmHg respectively. The postoperative effective orifice area in these two groups were respectively 1.5 =/-0.19 cm^2^ vs 1.1=/-0.5 cm^2^ (p 0.002). Among the 25 patients of the Soprano stented valve, 3 (12 %) came back in 6 months with New York Heart Association (NYHA) 3. The PPM of these patients was the cause of readmission in the Hospital required diuresis and supplementary treatment.

**Conclusions:**

Aortic valve replacement with Perceval aortic valves in geriatric patients with comorbidities and small aortic annulus seems to be an alternative, safe and “fast” intervention with excellent short and mid-term results which provides a better effective orifice area.

## Background

**Fig. 1:**
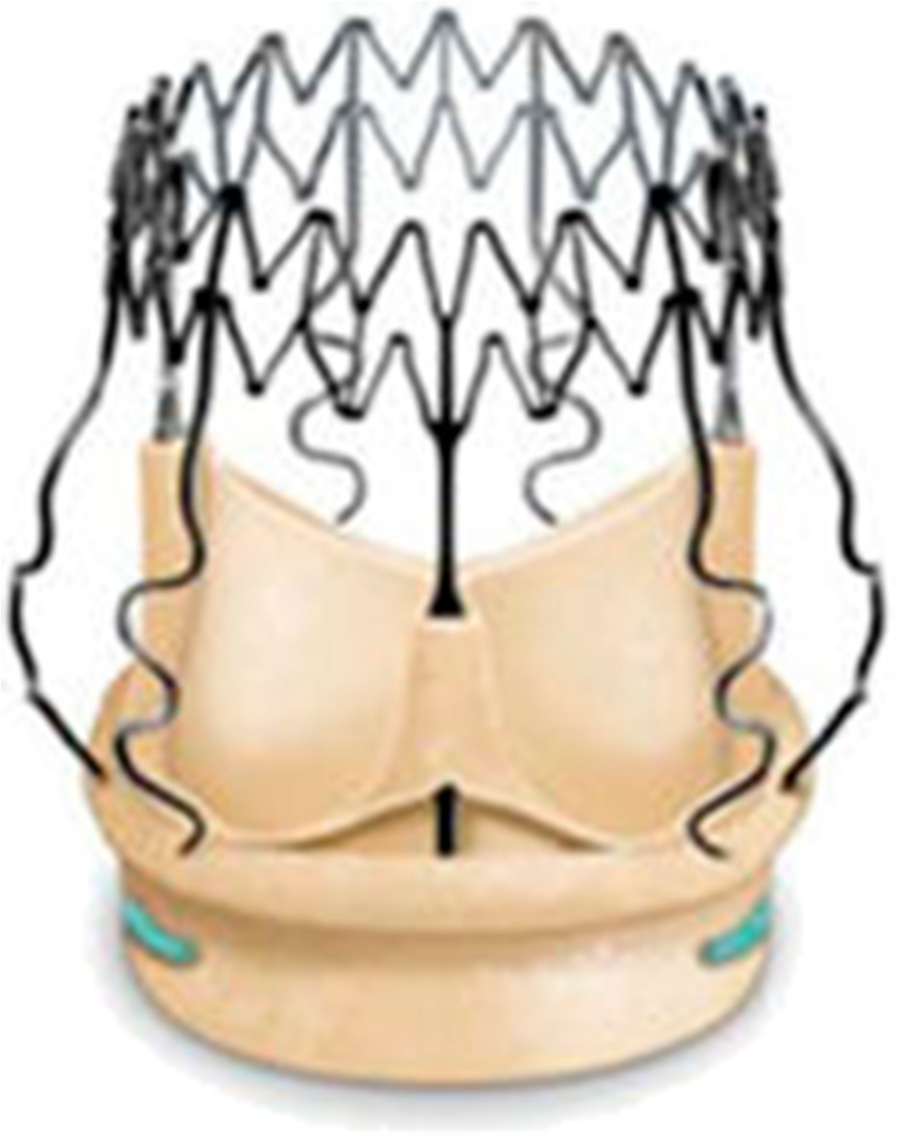
The Perceval S aortic valve is a bioprosthesis comprising a bovine pericardium tissue valve attached to a self-expanding anchoring device

**Fig. 2:**
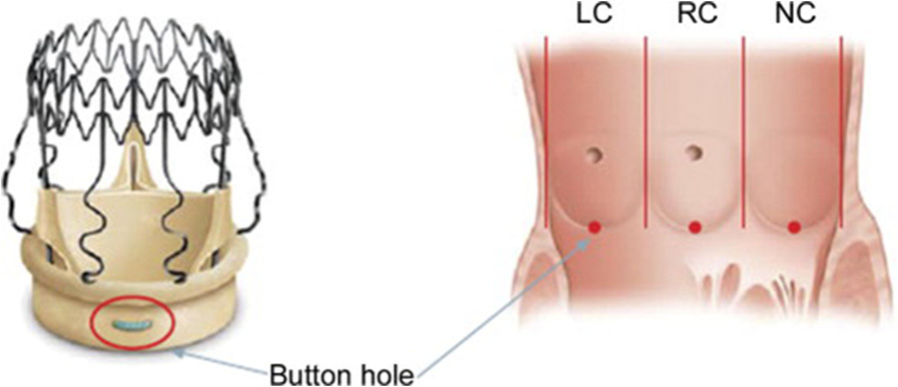
This device through its three button holes provides the correct positioning of the valve in the native aortic root

**Fig. 3:**
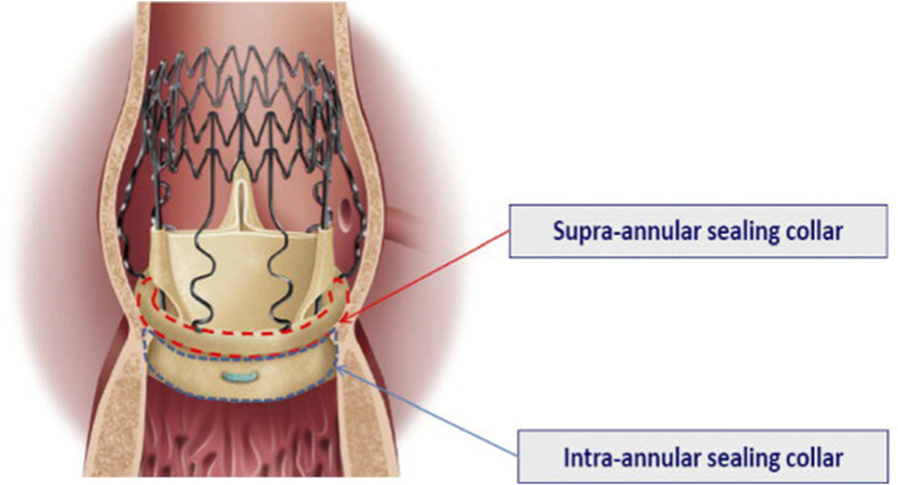
In order to minimize or avoid the paravalvular leakage, the Perceval S valve is designed with an intra–annular and a supra–annular sealing collar

**Fig. 4:**
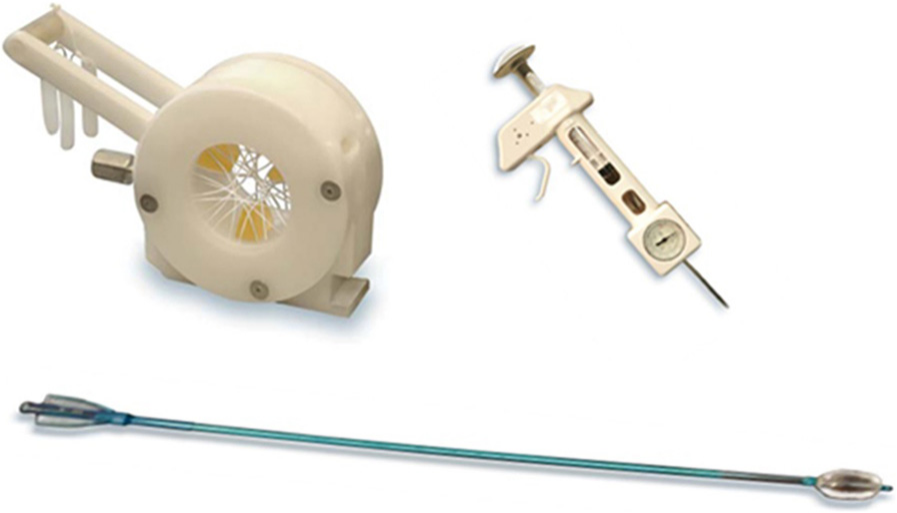
The prosthetic implant is supported by dedicated tools: crimping system, manometer and dilatation balloon

**Fig. 5:**
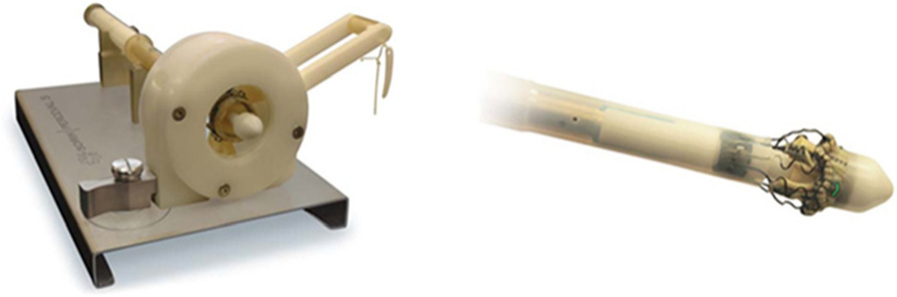
Prior to its implantation the prosthesis diameter is reduced to a suitable size, using the Perceval S collapsing tool, and then loaded on the Perceval S special holder

**Fig. 6:**
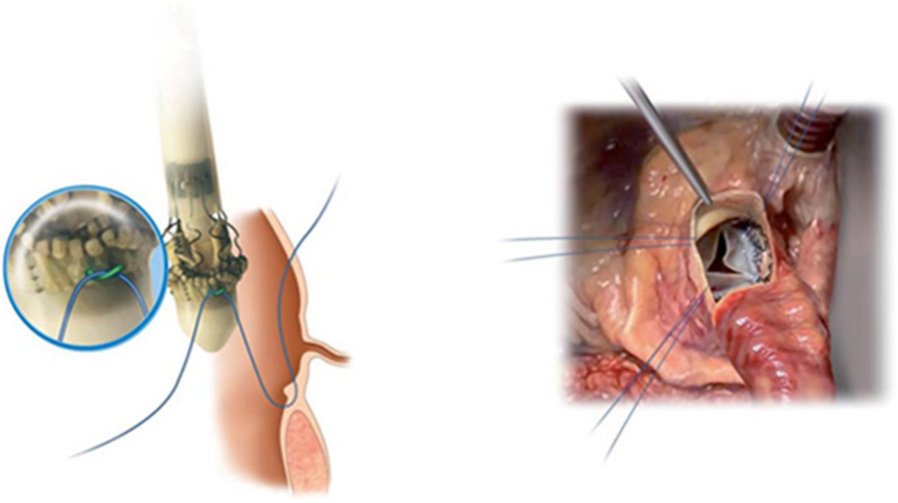
Using the three guides it performs in situ positioning of the valve. It is released in two steps: first the inflow ring is released at the native aortic annulus level and then, when proper positioning is verified, the complete prosthesis release is achieved

The sutureless Perceval bioprosthesis (LivaNova Biomedica Cardio Srl, Sallugia, Italy) was designed in order to obtain the hemodynamic benefits of the stentless valves without the increased difficulty in surgical implantation [1]. This valve is a bioprosthesis comprising a bovine pericardium tissue valve attached to a self-expanding anchoring device (Fig. 1), which has the dual role of supporting the bioprosthetic valve and offering fixation to the implantation site in the native aortic annulus [1]. As a result of the sutureless implant procedure, patients could benefit from reducing aortic cross-clamp time, with subsequent overall reduction of the surgical duration and reduction in related risks by avoiding passing the stitches through the calcified annulus and sutures knotting, with subsequent less risk of tearing the annulus and aortic wall or embolizing the systematic circulation [2]. In a small and calcified annulus it can be challenging to insert a stented valve and a significant residual gradient is frequently observed afterwards. Stentless valves are designed in order to overcome some of the disadvantages of the stented valves [1, 3, 4]. This device with its three button holes provides the correct positioning of the valve in the native aortic root (Fig. 2). In order to minimize or avoid the paravalvular leakage, the Perceval S valve is designed with an intra–annular and a supra–annular sealing collar (Fig. 3). This device is the ideal solution for elderly patients who require a rapid procedure and for patients with small aortic root which require root enlargement. As known, an aortic root enlargement (Nikcs-Nunez or Manougian technique) may be necessary in small annulus in order to avoid a “patients-prosthesis mismatch” [5]. This operation is challenging in elderly patients with comorbidities and heavily calcified aorta which a rapid intervention is necessary. The prosthetic implant is supported by dedicated tools: crimping system, manometer and dilatation balloon (Fig. 4). Prior to its implantation the prosthesis diameter is reduced to a suitable size, using the Perceval S collapsing tool, and then loaded on the Perceval S special holder (Fig. 5). After in situ positioning the valve is released in two steps: first the inflow ring is released at the native aortic annulus level and then, when proper positioning is verified, the complete prosthesis release is achieved (Fig. 6). After the implantation the Perceval S post-dilation balloon catheter is inflated inside the prosthesis at the inflow level to improve apposition by modeling the inflow ring on the native annulus [1, 6, 7]. In vitro accelerated fatigue tests were performed under normal and hypertensive conditions. These tests demonstrated that the whole device remains functional up to 900 Million cycles (more than 20 year of normal equivalent life). These results exceed the minimal ISO and FDA requirements and suggest a wide safety margin of the Perceval S bioprosthesis [1, 7]. We report our experience with the Perceval S bioprosthesis implanted onto 25 elderly with small aortic annulus and low BSA and we compared this group with 25 similar patients receiving conventional stented bioprosthesis. Our study is conducted in order to investigate two end points: effectiveness and performance so we will basically see its hemodynamic profile and its impact on effective orifice area other than the whole clinical outcome of the patients in the early postoperative period (within a month following surgery).

## Methods

In our study 25 sutureless self-anchoring, Perceval S, (LivaNova Biomedica Cardio Srl, Sallugia, Italy) valves are compared with 25 conventional biological stented prosthesis (soprano- Sorin Group) implanted onto similar characteristic patients with severe aortic stenosis. The study was conducted from January 2012 to June 2014. Patients were randomized divided into groups and they have previously consented for either method of surgical treatment. Randomization was done via a computerized assisted mathematic model. The inclusion criteria were defined as to see how the elderly patients with small aortic root and low BSA who are the real life difficult patients, will benefit from the use of a novel sutureless self-anchoring biological prosthesis. Limitations of this study were defined in a country with strict economic environment. The mean EuroSCORE II was 9.5 ± 3.5 in the Perceval group and 9.9 ± 3.6 in the conventional group. The BSA in m^2^ was 1.45 ± 1.2 and 1.78 ± 1.1. The rest patient’s characteristics are showing in the Table 1. The hemoglobin level was 33.3 g/L in the Perceval S group and 32.8 in the Soprano stented group preoperatively. Then, postoperatively, the hemoglobin was 28.6 in the Perceval S group and 28.8 in the second group. So, we did not find any statistically significate difference between the groups. All patients were treated with median full sternotomy, routine cannulation to the extracorporeal circulation with Edwards aortic cannula at the distal part of the ascending aorta and a two-stage venous cannula at the right atrium. Retrograde cardioplegia plus elective cardioplegia to the right coronary artery (ARC) was given in all patients. There was just one initial dose of cardioplegia given to all patients accompanying by local cooling with ice slush. No systematic extra cooling was required in our patients. The cross clamp was applied as distal as possible and aortotomy was performed approximately 2 cm above the sino-tubular junction (STJ). For the cases of conventional stented valve implantation an extension of the aortotomy towards the non-coronary sinus was performed. Pledged interrupted inverted stiches were met for the stented valve suturing. This is a prospective randomized study. From our study were excluded patients with previous cardiac surgery (redo operations), patients with aortic insufficiency, large aortic root (> or = 30 mm), sino-tubular junction periphery/height from the annulus > or = 1.3. Also, patients with BSA > or = 2 m^2^ and patients younger than 75 years old were excluded from our study.

**Table 1:** Preoperative patient’s characteristics and demographics

	SVP (25) sutureless valve	BVP (25) classic (soprano)
Number of patients	25	25
Age (mean)	80 ± 3.3	79 ± 4.1
Sex (**♀**/total)	15/25	11/25
Euro Score II	9.5 ± 3.5	9.9 ± 3.6
BSA (m^2^)	1.45 ± 1.2	1.78 ± 1,1
Stroke history	2/25 (8 %)	1/25 (4 %)
Preop rhythm	2/25 rbbb, 1/25 lbbb, 16/25 NSR, 1/25 A-F	3/25 rbbb, 2/25 A-F, 15/25 NSR
Concomitant CAD requiring CABG	1/25 (2 grafts)	2/25 (1 graft each)

*BSA* body surface area, *CAD* coronary artery disease, *CABG* coronary artery bypass graft

Ethical approval for this clinical study was obtained by our Hospital (General Hospital of Athens), Scientific Committee.

## Results

No structural prosthesis deterioration, valve thrombosis, or significant transvalvular aortic regurgitation occurred during the study period. There were no cases of tilting or migration once appropriately inserted during the entire study. This sutureless bioprosthesis appears to be ideal for patients with severe calcification of the aortic root and patients requiring concomitant procedures in whom a reduced bypass time is mandatory [1]. Cross clamp time and cardio-pulmonary bypass (CPB) time are reported at Table 2. All echo-cardiographic measurements were assessed by transthoracic echo. The annulus size was between 21 and 30 mm. We have implanted 18 perceval S valves size small, five medium and two large. In the conventional group we implanted 18 Soprano stented valves of the 21 mm and 4 Soprano stented valves of 20 mm. Postoperative EOA was 1.5 ± 0.3 cm^2^ in the Perceval group vs 1.1 ± 0.5 in the conventional group (p 0.002). The mean EOA index was 1.034 in the Perceval S group, while in the conventional group the EOA index was 0.617. This is the reason why three of the patients with Soprano stented valve came back in 6 months with New York Heart Association (NYHA) 3. The PPM of these patients was the cause of readmission in the Hospital required diuresis and supplementary treatment. This is the most important result coming out from our study. Operation time, CPB time and cross clamp time were significantly lower in the Perceval group (*p* < 0.001) as we could see in Table 2. These data are very important in cardiac surgery procedure and especially in elderly with comorbidities. The postoperative echo measurements were made within a month of period following surgery.

**Table 2:** Peroperative date and results

	SVP (25) sutureless valve	BVP (25) classic biological valve (soprano)	*P* value
Number of patients	25	25	
Preop. max gradient	88 ± 10.5	89 ± 12.5	
Postop. max gradient	23.5 ± 19.20 mmHg	24.5 ± 19.90 mmHg	0.670
Preop EOA	0.45 ± 0.19	0.47 ± 0.21	
Postop ΕΟΑ	1.5 ± 0.3 cm^2^	1.1 ± 0.5 cm^2^	0.002
Operation time	149.38 ± 15.22 min	206.64 ± 42.85 min	*p* < 0.001
CPB time	73.75 ± 8.12 min	120.36 ± 28.31 min	*p* < 0.001
Ischemia time	40 ± 5.50 min	86 ± 15.86 min	*p* < 0.001
Temporary postop pacing, permanent	15/25-3/25	2/25- 0/25	
Postoperative intubation time	6 ± 1.5 h	7 ± 1 h	
ICU stay	15 ± 3.5 h	16 ± 4 h	
Hospital stay	8 ± 1.5 days	7 ± 1.8 days	
Postop follow-up	8 ± 1.5 months	8 ± 1.8 months	
Death	0/25	1/25 arhythmia	

Comparison of the two groups of patients. *EOA* effective orifice area, *ICU* intensive care unit

## Conclusions

We studied the behavior of the Perceval S sutureless stentless bioprosthesis in patients with small aortic annulus, small BSA and older than 75 years. It seems that this is the target group of this valve. This group of patients with comorbidities and calcified aorta needs a rapid operation with minimal aortic manipulation. Age itself is not a contraindication to conventional surgery but comorbidities such as low ejection fraction, renal dysfunction and calcified aorta are major risk factor for mortality and morbidity [1, 8]. According the international bibliography [9], cross clamp time is an independent predictor of mortality and morbidity in low and high-risk cardiac patients. They found that prolonged aortic cross clamp time significantly correlated with worse clinical outcomes. The spectrum of complications included in-hospital mortality, prolonged hospitalization, prolonged ventilation, low cardiac output, higher requirements for blood transfusion and renal complications. In Perceval group patients, cross clamp time is significantly shorter than conventional group. This is an important advantage of this valve. Patients with low left ventricle ejection fraction (LVEF) are also candidates for this valve in order to implant rapidly an aortic valve without long ischemic time and consequently myocardial injuries. Due to the changing population demographics, the age of the patients presenting for AVR is also increasing [10]. A smaller-sized prosthetic valve may result in so-called patient-prosthesis mismatch (PPM). Therefore, different options have been proposed for patients with small aortic root presenting for AVR [5, 10]. Aortic root enlargement in elderly patients with heavily calcified aorta and comorbidities is a challenging operation with prolonged cross clump time and possible complications intra and postoperatively. In our opinion, old patients with renal dysfunction or history of cerebrovascular diseases may benefit from this kind of operation due to diminished operation time and less aortic manipulation. This valve may enable a broader application of minimally invasive AVR. Further longer-term experience is needed to determine the potential clinical benefits and durability of the Perceval S self-anchoring valve [10]. In case of valve malposition, there is the possibility of removal and re-implantation according the literature [11]. The valve implantation is possible with partial “j” sternotomy at the third or fourth intercostal space [12] in order to minimize the chest wall trauma and the risk of chest instability or infection. The studies and the international bibliography confirm the safety, efficacy, and ease of insertion of Perceval valves in elderly patients with small annulus [13–15]. As these valves do not need to be ‘sutured’, shorter cross-clamp and CPB times are possible. Moreover, due to the absence of a sewing ring, these valves are also almost ‘stentless’, with a greater valve EOA for any given size. This may therefore result in better hemodynamic even without the root enlargement [10]. According the literature [6], Perceval S valve could be implanted in elderly patients who require concomitant cardiac operation in order to minimise the operation time. Sutureless valves may be advantageous compared to transcatheter valve implantations as concomitant procedures other than percutaneous coronary artery angioplasty are not always possible in the latter [6, 15]. According our results, patients older than 75 years, with small aortic annulus and small BSA may benefit from a Perceval S aortic valve implantation. The limitation of our study is the small number of patients that were randomized into the two groups. However it is practically very difficult to study a large group of patients with the same characteristics in a single center. There is a large multicenter randomized trial going on and the results are expected with great interest. At the present time, the Perceval S prosthesis has been investigated in three clinical studies: 1. The “PERCEVAL TRIAL- Perceval S valve pilot trial-v10601”, 2. The “PERCEVAL Pivotal Trial – v10801”, 3. The “CAVALIER – Perceval S valve clinical trial for extended CE mark-TPS001” [7].
